# Cationic Hydroxyethyl Cellulose Nanocomplexes and RANK siRNA/Zoledronate Co-Delivery Systems for Osteoclast Inhibition

**DOI:** 10.3390/pharmaceutics16121623

**Published:** 2024-12-22

**Authors:** Sohyun Lee, Seoyeon Park, Tae-il Kim

**Affiliations:** 1 Department of Agriculture, Forestry and Bioresources, College of Agriculture and Life Sciences, Seoul National University, 1 Gwanak-ro, Gwanak-gu, Seoul 08826, Republic of Korea; hyonie99@snu.ac.kr (S.L.); dawnsy@snu.ac.kr (S.P.); 2 Research Institute of Agriculture and Life Sciences, Seoul National University, 1 Gwanak-ro, Gwanak-gu, Seoul 08826, Republic of Korea

**Keywords:** hydroxyethyl cellulose, nanocomplex, RANK siRNA, zoledronate, drug/gene co-delivery systems, osteoclast inhibition

## Abstract

Background/Objectives: In this study, HECP2k polymer, polyethylenimine2k (PEI2k)-modified hydroxyethyl cellulose (HEC) was utilized to form the nanocomplexes with receptor activator of nuclear factor k-B (RANK) siRNA and zoledronate (Zol) for osteoclast inhibition. HECP2k/(RANK siRNA + Zol) nanocomplexes prepared by simple mixing were anticipated to overcome the low transfection efficiency of siRNA and the low bioavailability of Zol. Methods: The characterization of both HECP2k/(pDNA + Zol) nanocomplexes and HECP2k/(RANK siRNA + Zol) nanocomplexes was performed. Results: The nanocomplexes were successfully formed even in the presence of Zol, showing about 200 nm sizes and about 20 mV of positive zeta potential values suitable for efficient cellular uptake. They also possessed high endosome buffering ability by PEI and Zol, suggesting the potential for efficient endosomal escape. It was found that the low cytotoxic nanocomplexes (>90% cell viability) displayed greater transfection efficiency than PEI25k and even HECP2k polyplexes. Finally, it was found by tartrate-resistant acid phosphatase (TRAP) assay and qPCR analysis that HECP2k/(RANK siRNA + Zol) nanocomplexes could inhibit the TRAP to about 50% value and another characteristic osteoclastic gene expression, increasing FAS gene expression to about 16 times higher than control and more efficiently (about 3 times and 5 times higher, respectively) than HECP2k/siRNA polyplexes and Zol only. Conclusions: HECP2k/(RANK siRNA + Zol) nanocomplexes formed by simple mixing showed great potential for inhibiting osteoclast differentiation and osteoclast activity, inducing the apoptosis via combinatorial effects of RANK siRNA and Zol.

## 1. Introduction

Osteoporosis is a metabolic disease characterized by bone mass reduction and bone structure deterioration [1,2]. Bone remodeling is regulated by interactions between two different cells, osteoclasts and osteoblasts [3]. Osteoclasts resorb the old bone tissue and remove the damaged tissue, whereas osteoblasts form new bones and grow existing bone tissues. If the remodeling system between the two cells fails in balance, it can lead to osteoporosis as osteoclast activity prevails [4].

Bisphosphonates, one of the most common drugs for treating osteoporosis, possess antiresorptive activity interfering with osteoclast function [5,6]. Having the configuration of a covalently linked P-C-P chain, the two side chains (R1, R2) determine the mechanism of each bisphosphonate. There are two categories of bisphosphonates according to the presence of N atom. Non-N-containing bisphosphonates that closely resemble pyrophosphates inhibit the aminoacylation of tRNA. However, their anti-resorptive potency is at the lower end of the scale compared to N-containing bisphosphonates, which inhibit protein prenylation by the blockade of farnesyl pyrophosphate synthase in osteoclasts [7]. However, it was reported that the single long-term usage of bisphosphonates can result in osteonecrosis of the jaw [8] and femoral fracture [9]. Recently, siRNA gene delivery has also gained a lot of attention in osteoporosis research [10]. siRNA for SOST gene encoding sclerostin that inhibits bone formation was loaded in polyethylenimine (PEI)-coated mesoporous silica nanoparticles with osteostatin, a parathyroid hormone-related protein and the synergistic effect of the two osteogenic agents was confirmed [11]. It was also reported that casein kinase-2 interacting protein-1 (CKIP-1) siRNA-based gene silencing with peptide-conjugated cationic liposomes could promote bone formation in osteogenic lineage cells [12].

The receptor activator of nuclear factor k-B (RANK) is located on the membrane of osteoclast precursor, inducing osteoclast differentiation by binding to RANK ligands (RANKL) secreted from osteoblast. RANKL binding to RANK leads to nuclear factor-κB (NF-κB) activation and its translocation to the nuclear. Subsequently, C-Fos expression increases, and its interaction with NFATc1 triggers the transcription of osteoclastogenic genes, leading to the expression of TRAP and cathepsin-K [13]. Therefore, many researchers are focusing on strategies to inhibit RANK expression in treating osteoporosis. The RANK siRNA delivery system combined with injectable bone-augmenting calcium phosphate cement was developed, showing the potential as bone augmentation materials [14]. The PEI/RANK siRNA complexes entrapped with nano-sized poly(lactic acid-co-glycolic acid) capsules were also reported, which reduced the RANK mRNA level and suppressed the osteoclast maturation [15].

In this study, RANK siRNA and zoledronate (Zol) were employed to inhibit the osteoclasts, using hydroxyethyl cellulose (HEC) derivative-based drug/gene co-delivery systems prepared by simple formulation for the first time. Zol is one of the potent bisphosphonate derivatives for inhibiting bone resorption of osteoclasts [16]. Imidazole moieties of Zol may contribute to endosome buffering effects, which can increase gene delivery efficiency [17,18]. Therefore, the co-delivery of these two anti-resorptive agents would enhance the efficacy of osteoclast inhibition. However, efficient carrier systems need to be developed because of their poor pharmacokinetics [19,20]. Here, HECP2k polymer was synthesized by conjugation of polyethylenimine2k (PEI2k) to HEC by reductive amination and utilized as delivery systems for osteoclasts inhibition, based on its low cytotoxicity and high transfection efficiency already reported in the previous study [21]. RANK siRNA and Zol with negative charges could form the nanocomplexes with cationic HECP2k polymer via electrostatic interaction by simple mixing without complicated chemical conjugation. It is generally thought that nanostructure-based drug delivery systems can load and stabilize diverse drug molecules via encapsulation or adsorption, targeting their delivery to cells and permitting controlled release. They also can exhibit high biosafety and offer tunable physicochemical properties [22,23]. Therefore, it is expected that these HECP2k-based nanocomplexes can overcome the poor pharmacokinetics of siRNA and Zol. Then, the physicochemical properties of the nanocomplexes, such as average sizes and zeta-potential values, were characterized, followed by verification of cytotoxicity, transfection and cellular uptake efficiency. Finally, TRAP assay and qPCR analysis were performed after the treatment of the nanocomplexes to osteoclasts to examine their osteoclast inhibition activity.

## 2. Materials and Methods

### 2.1. Materials

Hydroxyethyl cellulose (HEC), branched polyethylenimine 25k (PEI25k, 25 kDa), agarose, gel loading solution, ethidium bromide (EtBr), dimethyl sulfoxide (DMSO), MicroBCA™ protein assay kit, trypan blue, glycerol, and Ficoll^®^ 400 were all purchased from Sigma-Aldrich (St. Louis, MO, USA). Branched polyethylenimine 2k (PEI2k, 2000 Da) was purchased from Polysciences (Warrington, PA, USA). Sodium metaperiodate was purchased from Alfa-Aesar (Haverhill, MA, USA). Sodium tetrahydroborate and HPLC water were purchased from Duksan (Ansan, Republic of Korea). TAE buffer was purchased from Enzynomics (Daejeon, Republic of Korea). Dialysis membrane (MWCO 3.5 kDa) was purchased from Spectrum LABS (San Francisco, CA, USA). Formic acid, ethanol, hydrochloric acid, and Amicon^®^ Ultra-4 Centrifugal filters 3K were purchased from Merck (Darmstadt, Germany). Triazolyl blue tetrazolium bromide (MTT) was purchased from Gold Biotechnology (St. Louis, MO, USA). pDNA (pCN-Luci) was amplified by using Dyne DH5α Chemically Competent *E. coli* ver.2 (DYNEBIO, Sungnam, Republic of Korea). NucleoBond Xtra Maxi and NucleoSpin RNA extraction kit were purchased from Macherey-Nagel (Duren, Germany). Dulbecco’s Modified Eagle Medium (DMEM) and Dulbecco’s phosphate buffered saline (DPBS) were purchased from Cytiva (Marlborough, MA, USA). DMEM GlutaMAX™ (GMX), fetal bovine serum (FBS), penicillin-streptomycin (P/S), and trypsin-EDTA were purchased from Gibco (Waltham, MA, USA). α-Minimum Essential Medium (α-MEM) was purchased from WELGENE (Gyeongsan, Republic of Korea). Zoledronic acid monohydrate (Zol) was purchased from TCI (Tokyo, Japan). Recombinant Murine sRANK Ligand was purchased from PeproTech (Cranbury, NJ, USA). TRACP & ALP double-stain kit, TRACP & ALP Assay kit, PrimeScript™ RT Reagent kit, and TB Green^®^ Premix Ex Taq™ II were purchased from Takara Bio (Kusatsu, Japan). The Luciferase assay system and reporter lysis buffer were purchased from Promega (Madison, WI, USA). Carbon film 300 mesh copper TEM grid was purchased from Electron Microscopy Sciences (Hatfield, PA, USA). Quant-iT™ PicoGreen™ dsDNA assay kit, Ambion™ Nuclease free water, and YOYO-1 iodide were purchased from Invitrogen (Waltham, MA, USA). RANK siRNA (5′-GCGCAGACUUCACUCCAUAUU-3′) and other primers were purchased from Bioneer (Daejeon, Republic of Korea). All other chemicals were purchased and used without any further purification.

### 2.2. Synthesis and Characterization of HECP2k

Delivery carrier polymer, HECP2k, was synthesized by reductive amination of oxidized HEC (oxHEC) with PEI2k and the synthesis of HECP2k was confirmed according to the previous report [21]. HEC was oxidized in a water solution by sodium metaperiodate (NaIO_4_) with one molar equivalent amount of HEC sugar unit. After 2 h of reaction at 25 °C in the dark, oxHEC was obtained by dialysis (MWCO 3.5 kDa) against ultra-pure water for 48 h, followed by lyophilization. Then, oxHEC/water solution was mixed with polyethylenimine (PEI2k)/water solution (oxHEC:PEI2k = 1:10, *w*/*w*) for the conjugation reaction of 24 h at 25 °C in the dark. Subsequently, an excessive amount of sodium tetrahydroborate (NaBH_4_) was added to the mixture solution, and the reduction reaction was performed for 24 h at 25 °C in the dark. The final product, HECP2k, was obtained by dialysis against ultra-pure water for 72 h, followed by lyophilization.

The synthesis of HECP2k polymer was confirmed by 1H NMR (600 MHz AVANCE 600, Bruker, Billerica, MA, USA) in D_2_O with a concentration of 10 mg/mL. Then, the molecular weight of HECP2k was examined with gel permeation chromatography (GPC) (YL-9100 HPLC System, Youngin Chromass, Anyang, Republic of Korea) with Ultrahydrogel 250 column from Waters (Milford, MA, USA). The concentration of the polymer was 10 mg/mL with 1% formic acid as an eluent. Poly(ethylene glycol) with various molecular weights were used as standard molecules.

### 2.3. Formation of the HECP2k Nanocomplexes

Zoledronate (Zol)-containing HECP2k nanocomplexes were prepared by encapsulation method [18]. First, Zol with various amounts (Zol:pDNA or siRNA = 0.2~10:1, *w*/*w*) and pDNA or siRNA were mixed together for 30 min. The mixture solution was carefully added to the HECP2k solution (HECP2k:pDNA or siRNA = 50:1, *w*/*w*). After 30 min of incubation, HECP2k/(pDNA + Zol) or HECP2k/(siRNA + Zol) nanocomplexes were formed, respectively.

### 2.4. pDNA Condensing Ability Analysis of the Nanocomplexes

The effect of Zol loading on the pDNA or siRNA condensing ability of HECP2k was examined by agarose gel electrophoresis. All HECP2k/(pDNA + Zol) nanocomplex or HECP2k/(siRNA + Zol) nanocomplex samples containing various amounts of Zol were prepared in HEPES buffer (pH 7.4) and incubated for 30 min, as mentioned above. Then, the samples were loaded in 0.7% agarose gel containing EtBr solution (0.5 μg/mL) and electrophoresed for 12 min at 80 V using Mupid-2plus^®^ (Takara Bio, Kusatsu, Japan). Visualization of pDNA bands was performed by GelDoc^TM^XRS+gel documentation system (Bio-Rad, Hercules, CA, USA).

The effect of Zol loading was further quantified by PicoGreen assay [24]. First, HECP2k/(pDNA + Zol) nanocomplex samples (0.5 μg pDNA, Zol:HECP2k:pDNA = 0.2~5:50:1, *w*/*w*/*w*) were prepared. HECP2k polyplex samples (0.5 μg pDNA, HECP2k:pDNA = 0.1~50:1, *w*/*w*) were also prepared as controls. Then, 100 μL of sample solutions were loaded in a black opaque 96-well plate, and 100 μL PicoGreen reagent solution was added to each well. After 4 min of incubation in dark condition, the fluorescence of each sample was measured (Ex: 480 nm, Em: 520 nm) using a microplate reader (Synergy H1, BioTek, Winooski, VT, USA). The results were presented as relative fluorescence of samples to free pDNA. All the measurements were performed in triplicate.

### 2.5. Characterization of the Nanocomplexes

The Z-average sizes and zeta potential values of HECP2k/(pDNA + Zol) nanocomplexes were measured by Zetasizer (Nano ZS90, Malvern Instruments, Malvern, UK). After the preparation of complex solutions, as mentioned above, the measurement was performed 3 times. HECP2k/(siRNA + Zol) nanocomplexes containing RANK siRNA were also formed and examined in the same manner as well.

The morphology of HECP2k/(pDNA + Zol) nanocomplexes was observed with energy-filtering transmission electron microscopy (EF-TEM, LIBRA 120, Carl Zeiss, Oberkochen, Germany). 10 μL of complex solutions (2 μg pDNA) were loaded on a copper carbon film 300 mesh (CF300-Cu-50, Electron microscopy science, Hatfield, PA, USA) for 1 min. After blotting away the excess solution on the grid with filter paper, the grid was stained for 5 s with 2% uranyl acetate solution. After blotting the grid away again, the images were visualized with an accelerating voltage of 120 kV.

### 2.6. Zol Loading Confirmation of HECP2k/(pDNA + Zol) Nanocomplexes

Zol loading in complex particles was confirmed by measuring the absorbance of Zol after filtration [18]. HECP2k/(pDNA + Zol) nanocomplexes (Zol:HECP2k:pDNA = 20:50:1, *w*/*w*/*w*) containing 2 μg of pDNA were prepared. After 30 min of incubation, the complex solution was centrifuged with Amicon^®^Ultra-4 Centrifugal filters (3K) at 4000 rpm and 4 °C for 30 min. The filtrate was transferred to a quartz cell, and the absorbance of Zol (200~600 nm) was measured with a UV-VIS spectrophotometer (Optizen UV2120, Duksan Mecasys, Seoul, Republic of Korea).

### 2.7. Endosome Buffering Capacity of HECP2k/(pDNA + Zol) Nanocomplexes

Acid-base titration was performed to examine the endosome buffering capacity of HECP2k/(pDNA + Zol) nanocomplexes [24]. PEI25k and HECP2k polymers were used as controls. 5 mg of each polymer was dissolved in 5 mL of aqueous NaCl solution (0.1 M), and pH was adjusted to 11 using 0.1 M NaOH. In the case of the complexes, 200 μL of pDNA (0.5 mg/mL) was used for complexation. PEI25k polyplexes (PEI25k:pDNA = 1:1, *w*/*w*), HECP2k polyplexes (HECP2k:pDNA = 50:1, *w*/*w*), and HECP2k/(pDNA + Zol) nanocomplex (Zol:HECP2k:pDNA = 2:50:1, *w*/*w*/*w*) were prepared. Then, the solutions were titrated from pH 11 to pH 3 by dropping 5 μL of 0.1 M HCl. For the titration of Zol, 1 mL of Zol solution (0.01 M) was mixed with 4 mL of NaCl solution. Then, the pH of the mixture was adjusted to pH 3 using 1 M HCl solution. By dropping 5 μL of 0.1 M NaOH solution, the Zol solution was titrated to pH 11. The pH change of the solutions was measured by a pH meter (SevenEasy pH meter S20, Mettler-Toledo, Columbus, OH, USA).

### 2.8. Cell Culture

Mouse leukemic monocyte-macrophage cell line (RAW264.7, origin: mouse monocyte, macrophage, Korean Cell Line Bank) and human cervical adenocarcinoma cell line (HeLa, origin: human cervix, uterine, Korean Cell Line Bank) were used for in vitro experiments. Cells were cultured in a medium supplemented with 10% FBS and 1% penicillin/streptomycin (DMEM for RAW264.7, DMEM+GlutaMAX^TM^ for HeLa cells). All cells were maintained in a humidified atmosphere containing 5% CO_2_ at 37 °C.

### 2.9. MTT Assay

The cytotoxicity of Zol, HECP2k polymer, and the nanocomplexes was investigated using a MTT assay on HeLa and RAW264.7 cells [21]. The cells were seeded on a 96-well cell culture plate at a density of 1 × 10^4^ cells/well. After 24 h of incubation, the cells achieved 70~80% confluency. For the cytotoxicity of HECP2k polyplexes (HECP2k:pDNA = 50:1, *w*/*w*) and HECP2k/(pDNA + Zol) nanocomplexes (Zol:HECP2k:pDNA = 0.2~5:50:1, *w*/*w*/*w*), each sample was prepared with 0.1 μg of pDNA/well. Then, 100 μL of sample solutions (serum-free media) with various concentrations (0~100 μg/mL) were treated in each well. After 4 h of incubation, the media were changed to fresh media (10% FBS). After another 24 h of incubation, 25 μL of MTT solution (2 mg/mL in DPBS) was added to each well for 2 h at 37 °C. The medium was aspirated off, and 150 μL of DMSO was added to each well to dissolve the purple formazan crystal formed by the cell metabolism. The absorbance was measured at 570 nm using a microplate reader. The results were presented as relative cell viability (% RCV, percentage values to untreated cell viability).

### 2.10. Luciferase Transgene Expression Assay

Luciferase transgene expression assay was used to examine the transfection efficiency of HECP2k/(pDNA + Zol) nanocomplexes in HeLa and RAW264.7 cells [21]. Cells were seeded on a 24-well cell plate at a density of 5 × 10^4^ cells/well and incubated for 24 h. After 70~80% of confluency was achieved, the medium of each well was exchanged with fresh medium (serum-free or 10% serum-containing medium). Then, 50 μL of each sample with various Zol weight ratios was treated in the well (0.5 μg pDNA/well). PEI25k was used as a positive control. After 4 h of incubation, the medium was exchanged with fresh medium with 10% FBS, followed by 48 h of further incubation. Then, each medium was removed, and cells were rinsed with DPBS and lysed with 120 μL of reporter lysis buffer, followed by one cycle of freeze-thawing. The cell lysates were scrapped and centrifuged for 10 min at 14,000 RCF and 4 °C. 20 μL of supernatant was added to each well in a transparent 96-well plate. The luminescence was measured with a microplate reader, dispensing 100 μL of the luciferase assay buffer. For the quantification of the protein in the supernatant, a MicroBCA™ protein assay kit was utilized. 130 μL of ultra-pure water, 150 μL of BCA reagent, and 20 μL of the supernatant were added to a 96-well plate. Then, the plate was incubated at 37 °C for 2 h. Using a microplate reader, the absorbance was measured at 562 nm. Final protein-normalized luciferase transgene expression efficiency was presented as a relative light unit (RLU)/mg protein.

### 2.11. Serum Stability Test

Serum stability of HECP2k/(pDNA + Zol) nanocomplexes was determined by transfection in RAW264.7 cells. Cells were seeded on a 24-well cell plate at a density of 5 × 10^4^ cells/well and incubated for 24 h at 37 °C. After 70~80% of confluency was achieved, the medium of each well was exchanged with 450 μL of medium containing 10% or 30% FBS. Then, 50 μL of each complex solution (0.5 μg pDNA) was treated in the well. PEI25k was used as a control. After 4 h of incubation, the following luciferase transgene expression assay was performed as described above.

### 2.12. Cellular Uptake

For the estimation of cellular uptake of the HECP2k/(pDNA + Zol) nanocomplexes, flow cytometry was utilized [21]. RAW264.7 cells were seeded on a 6-well cell culture plate at a density of 2 × 10^5^ cells/well, respectively and further incubated for 24 h to achieve 70~80% confluency. pDNA was labeled with YOYO-1 iodide (1 dye per 50 base pairs of nucleotides). PEI25k polyplex, HECP2k polyplex (HECP2k:pDNA = 50:1, *w*/*w*), and HECP2k/(pDNA + Zol) nanocomplex (Zol:HECP2k:pDNA = 2:50:1, *w*/*w*/*w*) samples were prepared and treated to each well containing serum-free medium for 4 h. Then, the medium was removed, and the cells were rinsed with ice-cold DPBS twice. 1 mL of trypan blue solution (1 mg/mL) was treated to quench the fluorescence of the cell surface-bound polyplexes for 5 min in the dark. After rinsing again with ice-cold DPBS three times, the cells were detached from the plate and re-suspended in DPBS. The cellular uptake of fluorescence-labeled polyplexes was examined by using a BD Accuri C6 flow cytometer (BD Biosciences, Franklin Lakes, NJ, USA) at a minimum of 1 × 10^4^ cells gated per sample. The analysis was performed by using BD Accuri C6 (Version 1.0.264.21).

### 2.13. Osteoclast Differentiation

Osteoclast differentiation was conducted by treating RANKL (Receptor activator of nuclear factor-kappa B Ligand) to RAW264.7 cell [25]. RAW264.7 cells were seeded on a 6-well cell culture plate or 24-well cell culture plate at a density of 4 × 10^4^ cells/well or 1 × 10^4^ cells/well, respectively, in α-MEM (2.5% FBS and 1% penicillin/streptomycin). After 24 h of incubation, RANKL was treated three times every two days (50 ng/mL) for osteoclast differentiation.

### 2.14. Osteoclast Activity Analysis by TRAP Assay

A TRAP assay was conducted to examine the osteoclast differentiation using a TRACP & ALP double-stain kit, according to the manufacturer’s protocol. The differentiated cells were rinsed with 1 mL of DPBS and fixed with 1 mL of fixation base solution. After 5 min of incubation at 25 °C, the solution was aspirated off, and the cells were rinsed with DPBS twice. Then, the cells were treated with the substrate solution containing sodium tartrate (1 mL/well) for 45 min at 37 °C. After rinsing with water, glycerol was added to prevent the cells from drying. The cells were observed by using a microfilm camera (Olympus CKX41 Microscope, Tokyo, Japan) with the CellSense program.

The quantification of osteoclast activity was performed using the TRACP & ALP Assay kit, according to the manufacturer’s protocol. The cells were rinsed with DPBS and treated with 100 μL of extraction solution for lysis. Then, 50 μL of cell lysate solution and 50 μL of substrate solution were mixed in a 96-well plate. After 1 h of incubation at 37 °C, the reaction was stopped by adding 25 μL of NaOH (0.5 N) solution to each well. Then, the absorbance was measured at 405 nm after the color formation.

### 2.15. Gene Expression Analysis by Reverse-Transcription Polymerase Chain Reaction (RT-PCR)

Several gene expression profiles were analyzed by RT-PCR. RNA was extracted with a Nucleospin^®^ RNA extraction kit, according to the manufacturer’s protocol. Osteoclast cells were rinsed with 1 mL of DPBS twice, and a lysis buffer was treated on the cells to be harvested with a cell scraper. After the extraction, the total amount of mRNA was measured with a Take3 micro-volume plate (Synergy H1, BioTek, Winooski, VT, USA). cDNA was synthesized by using the PrimeScript RT reagent kit. Quantitative analysis of mRNA was performed by StepOne real-time PCR machine (Applied Biosystems, Waltham, MA, USA) with SYBR Premix Ex Taq II, the prepared cDNA, and primers. Table S1 shows the primer sequences used for RT-PCR. Briefly, the samples were denatured at 95 °C for 30 s with 40 cycles of PCR. A set of one cycle consisted of denaturation (95 °C, 5 s), annealing (55 °C, 30 s), and polymerization (72 °C, 30 s). The gene expression levels were normalized with the GAPDH gene, and the 2^−△△CT^ method was used [26].

### 2.16. Inhibition Analysis of Osteoclast Differentiation by TRAP Assay

To confirm the ability of HECP2K/(siRNA + Zol) nanocomplexes for inhibiting osteoclast differentiation, the nanocomplexes (Zol:HECP2k:siRNA = 2:50:1, *w*/*w*/*w*) were treated to the cells and incubated for further TRAP assay. RAW264.7 cells were seeded and treated with RANKL for differentiation, as above. On the 5th day of differentiation, the cells were treated with HECP2k, Zol, RANK siRNA, and HECP2K/(siRNA + Zol) nanocomplexes, respectively. The final concentration of siRNA was 5 nM. On the 7th day of incubation, a TRAP assay was conducted to confirm the osteoclast differentiation inhibiting ability of HECP2K/(siRNA + Zol) nanocomplexes.

### 2.17. Osteoclast Apoptosis Analysis by qPCR

The expression of the FAS gene was quantified to examine the ability of HECP2K/(siRNA + Zol) nanocomplexes in osteoclast apoptosis. The differentiated osteoclast cells were cultured and treated with HECP2K/(siRNA + Zol) nanocomplexes and equal amounts of HECP2k, Zol, and siRNA. The final concentration of RANK siRNA was 5 nM. With the mRNA quantification process as mentioned above, FAS gene expression was measured by qPCR. Non-treated osteoclasts were used as controls.

### 2.18. Osteoclast Inhibition Analysis by qPCR

RANK, NFATc1, and Cathepsin-K gene expression were analyzed by qPCR, respectively, in order to examine the inhibiting ability for osteoclast differentiation of HECP2K/(siRNA + Zol) nanocomplexes. RAW264.7 cells were seeded and treated with RANKL for differentiation, as above. HECP2K/(siRNA + Zol) nanocomplexes were treated in the cells on the 5th day of cell culture. With the mRNA quantification process, RANK, NFATc-1, and cathepsin-K gene expression were analyzed by qPCR. Non-treated osteoclasts were used as controls.

## 3. Results and Discussions

### 3.1. Synthesis and Characterization of HECP2k

Oxidation of HEC with sodium metaperiodate was conducted to convert the vicinal diols of HEC repeating units to aldehyde groups, cleaving the C-C bonds of vicinal diols. Oxidized HEC (oxHEC) was reacted with primary amine groups of PEI2k by Schiff base formation. As the imine bond is unstable, further reduction with sodium tetrahydroborate was performed to convert imine bonds to stable C-N bonds. The synthesis of HECP2k was identified by ^1^H NMR (Figure S1A) and GPC (Figure S1B) analysis. Proton peaks of HEC and PEI2k were observed at δ 3.15–4.30 and δ 2.35–3.05, respectively and a single monodisperse GPC peak was observed (M_w_ =15.8 kDa, PDI =2.20), confirming the synthesis of HECP2k.

### 3.2. Formation and Characterization of the Nanocomplexes

It was reported that the complexation of pDNA and Zol with cationic polymers could be carried out in two different ways [18]. First, the polymer is mixed with pDNA to form positively charged polyplexes, and the polyplex solution is dropped into Zol solution, which leads to the formation of Zol-coated polyplexes. These polyplexes showed large size and wide size distribution. Therefore, the second method was selected for the formation of stable HECP2k/(pDNA + Zol) nanocomplexes with proper size. The mixture solution of pDNA and Zol was dropped into the cationic HECP2k solution, forming nanocomplexes. In the previous study [21], HECP2k showed the highest cellular uptake, transfection, and gene silencing efficiency at a weight ratio of 50. Therefore, the weight ratio of HECP2k to pDNA or siRNA was determined to be 50 for the formation of nanocomplexes in all subsequent experiments. In most cases, the delivery of bisphosphonate has been accompanied by chemical reactions via covalent bond formation using various organic solvents or even complicated emulsification methods [27,28]. However, this simple mixing fabrication of HECP2k/(pDNA + Zol) nanocomplexes via electrostatic interaction is a less time and energy-consuming process.

**Figure 1:**
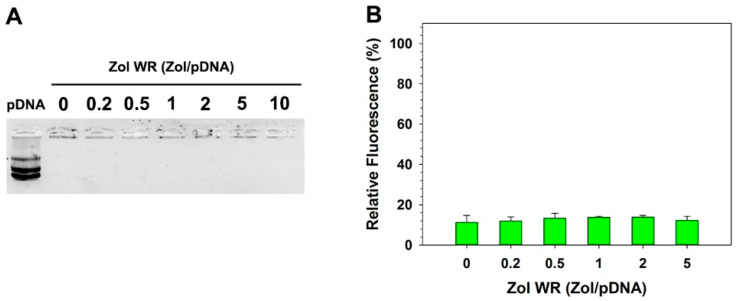
(**A**) Agarose gel electrophoresis result of HECP2k/(pDNA + Zol) nanocomplexes. (**B**) PicoGreen assay result of HECP2k/(pDNA + Zol) nanocomplexes (Zol:HECP2k:pDNA = 0~5:50:1, *w*/*w*/*w*).

The formation of stable HECP2k/(pDNA + Zol) nanocomplexes was assessed by agarose gel electrophoresis. Figure 1A showed no migration of pDNA bands from all complexes regardless of Zol weight ratios (up to 10), indicating that pDNA complexation with HECP2k was successful even in the presence of negatively charged Zol. The formation of stable HECP2k/(siRNA + Zol) nanocomplexes was also examined by agarose gel electrophoresis (Figure S2A). Like HECP2k/(pDNA + Zol) nanocomplexes, siRNA was efficiently complexed by HECP2k in the presence of Zol, too.

Zol’s effect on pDNA condensation of HECP2k was also examined using a PicoGreen assay. PicoGreen assay quantitates DNA by emitting fluorescence when the dye specifically binds to double-stranded DNA. As shown in Figure 1B, all fluorescence values of HECP2k/(pDNA + Zol) nanocomplexes were as low as that of HECP2k polyplexes (Zol WR = 0), meaning the formation of compact polyplexes. This is consistent with agarose gel data and indicates that pDNA was compactly condensed by HECP2k, implying the presence of Zol did not affect the pDNA binding ability of HECP2k.

### 3.3. Average Size and Zeta Potential Measurement of the Nanocomplexes

**Figure 2:**
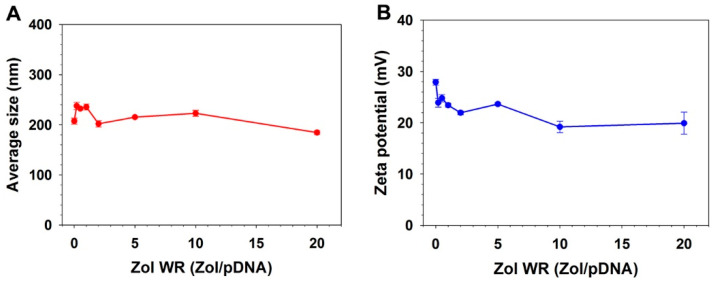
(**A**) Average sizes and (**B**) Zeta potential values of HECP2k/(pDNA + Zol) nanocomplexes with various weight ratios of Zol (Zol:HECP2k:pDNA = 0~20:50:1, *w*/*w*/*w*).

Z-average size and zeta potential of HECP2k/(pDNA + Zol) nanocomplexes were measured by Zetasizer. The average size measured by Zetasizer here means the hydrodynamic diameter, unlike the size measured by TEM. About 200 nm size was observed for HECP2k/(pDNA + Zol) nanocomplexes at all Zol weight ratios, which is suitable for efficient cellular uptake and endocytosis (Figure 2A). It means the formation of stable nanocomplexes even in the presence of Zol. The Zeta potential value of the complexes gradually decreased from about 30 to 20 mV as the Zol weight ratio increased, probably due to the negatively charged phosphate group of Zol (Figure 2B). The average size and zeta potential value of HECP2k/(siRNA + Zol) nanocomplexes using RANK siRNA were also measured, and the nanocomplexes showed 140~180 nm sizes (Figure S2A) and 17~24 mV zeta potential values (Figure S2B), meaning the formation of stable nanocomplexes in the presence of Zol, too. The PDI values of the nanocomplexes are presented in Table S2, and they range from 0.135 to 0.25, suggesting the formation of monodisperse complexes.

### 3.4. Morphology Observation by TEM

**Figure 3:**
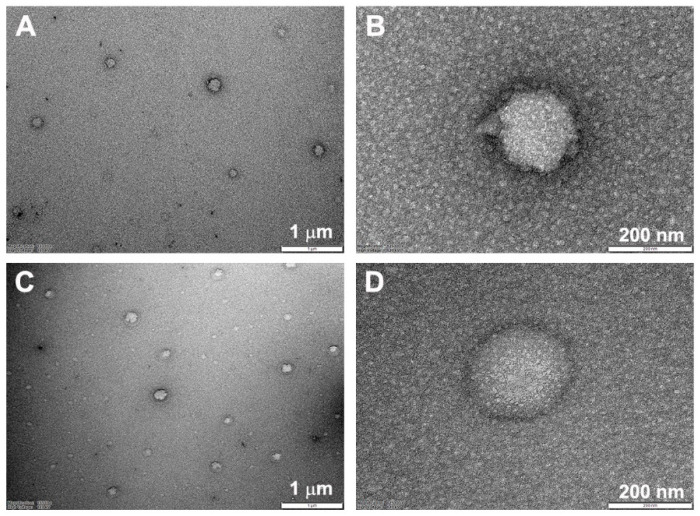
TEM images of HECP2k polyplexes (**A**,**B**) and HECP2k/(pDNA + Zol) nanocomplexes (**C**,**D**) (Zol:HECP2k:pDNA = 2:50:1, *w*/*w*/*w*).

The morphology of HECP2k polyplexes and HECP2k/(pDNA + Zol) nanocomplexes was observed using TEM. As shown in Figure 3, they both displayed uniform and spherical shapes, similar to each other. Therefore, it was thought that the presence of Zol did not affect the characteristics of complex morphology as well. The complexes smaller than 200 nm were also observed. It is usually thought that the size of the nanocomplexes measured by TEM would be smaller than that measured by Zetasizer due to the shrinkage of the nanocomplexes in the dry TEM condition.

The morphology of HECP2k/(siRNA + Zol) nanocomplexes was also observed using TEM. As shown in Figure S3, they also formed spherical particles with sizes ranging from 170~270 nm.

### 3.5. Zol Loading Ability of HECP2k/(pDNA + Zol) Nanocomplexes

The Zol loading ability of HECP2k/(pDNA + Zol) nanocomplexes was examined by measuring the Zol absorbance of the complexes after filtration. As shown in Figure S4A, Zol solution and filtered Zol solution showed similar absorbance peaks, which displayed maximum values at 215 nm. This means that Zol can penetrate through the Amicon tube membrane. If Zol could not be complexed by HECP2k polymer, the absorbance peak of unloaded Zol in the filtrate would be detected at 215 nm. However, even with large amounts of zoledronate (Zol WR = 20), the complex did not exhibit any absorbance at 215 nm. The absorbance at around 200 nm for filtered nanocomplexes comes from free HEC polymer. In addition, the absorbance of Zol at 215 nm after filtration of the HECP2k/(siRNA + Zol) nanocomplexes (Zol:HECP2k:siRNA = 20:50:1, *w*/*w*/*w*, 2 μg/mL siRNA) was measured and the loading efficiency was calculated based on the prepared calibration curve (Figure S4B). The absorbance of Zol in the filtrate was 0.0379, and the loading efficiency was 95.4%. This result supports that Zol can be loaded in the nanocomplexes without any significant loss, even in the presence of pDNA when complexed with HECP2k, showing high loading efficiency.

### 3.6. Endosome Buffering Capacity Measurements

Upon the uptake of the nanocomplexes into the cell by endocytosis, the nanocomplexes should escape from endosomes before the lysosomal degradation. Acid-base titration was conducted to verify the endosome buffering capacity of HECP2k/(pDNA + Zol) nanocomplexes. It was revealed that Zol showed buffering capacity in Figure S5A, as expected. Figure S5B shows that HECP2k/(pDNA + Zol) nanocomplexes possessed higher endosome buffering capacity than HECP2k polyplexes. Based on the titration curve, it was calculated that the nanocomplexes containing Zol possessed about 75% buffering capacity of equivalent Zol in addition to their own buffering capacity. Therefore, it was confirmed that Zol having imidazole and phosphate groups would contribute to the endosome buffering ability of the nanocomplexes without being inhibited upon complexation and expected that higher buffering capacity would lead to efficient endosomal escape of the nanocomplexes, increasing the overall gene delivery efficiency. Therefore, it was thought that Zol would function not only as a drug itself for inhibiting osteoclasts but as an endosome buffering agent for enhancing gene delivery simultaneously.

### 3.7. Cytotoxicity Measurements

**Figure 4:**
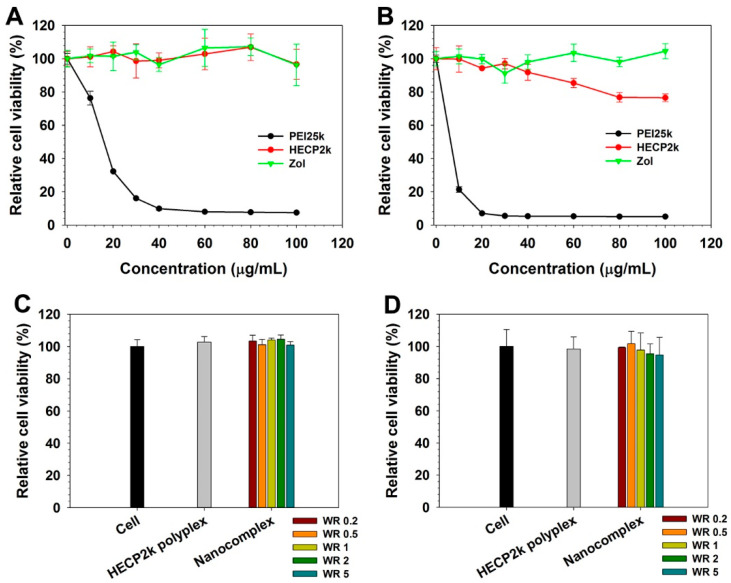
MTT assay results of HECP2k polymer and Zol on (**A**) HeLa and (**B**) RAW264.7 cells. MTT assay results of HECP2k/(pDNA + Zol) nanocomplexes on (**C**) HeLa and (**D**) RAW264.7 cells. WR means the weight ratio of Zol (Zol/pDNA) in the nanocomplexes. PEI25k and HECP2k polyplexes were used as controls.

Cytotoxicity of HECP2k and Zol was assessed by measuring cell metabolic activity with MTT assay (Figure 4A,B). In HeLa and RAW264.7 cells, PEI25k polymer showed well-known high cytotoxicity even at low concentrations. However, HECP2k and Zol exhibited relatively high cell viability (>80%) throughout all concentrations. This result demonstrates that HECP2k and Zol possess low cytotoxicity. Cytotoxicity of HECP2k/(pDNA + Zol) nanocomplexes was further examined by MTT assay (Figure 4C,D). The nanocomplexes also showed high cell viability (>90%) for all cell lines even though they are Zol-containing formulations, confirming their low cytotoxicity.

### 3.8. Transfection Efficiency Measurements

**Figure 5:**
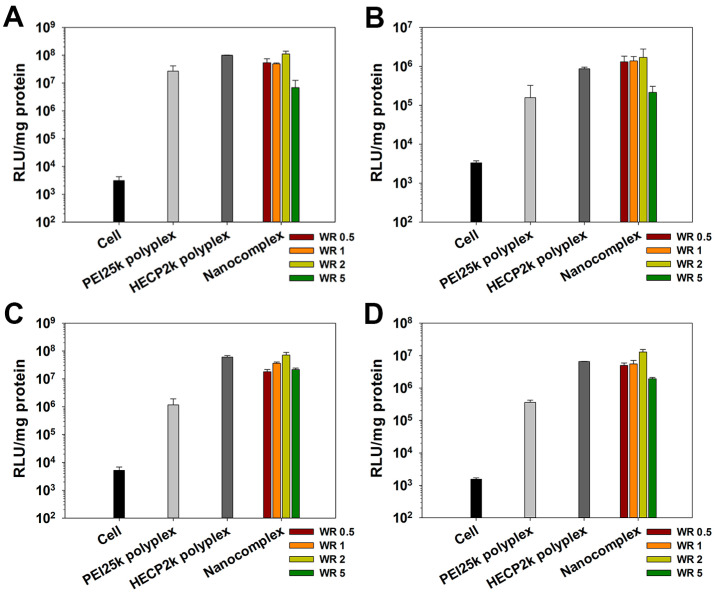
Transfection efficiency of HECP2k/(pDNA + Zol) nanocomplexes with various Zol weight ratios (WR) on HeLa (**A**,**B**) and RAW264.7 cells (**C**,**D**). Transfection experiments were performed in serum-free conditions (**A**,**C**) and serum conditions (**B**,**D**).

Transfection efficiency of HECP2k/(pDNA + Zol) nanocomplexes containing various amounts of Zol was examined in HeLa and RAW264.7 cells by luciferase transgene assay. PEI25k and HECP2k polyplexes were used as controls. As previously reported [21], HECP2k polyplexes showed higher transfection efficiency than PEI25k polyplexes in both serum-free and serum conditions. Interestingly, the nanocomplexes showed similar or even higher efficiency than HECP2k polyplexes (Figure 5). The transfection efficiency of the nanocomplexes containing Zol with a weight ratio of 2 was the highest in all conditions, about 2 times higher than that of HECP2k polyplexes, which suggests that Zol may facilitate the endosomal escape due to the endosome buffering ability of imidazole groups [29,30]. Therefore, the Zol weight ratio was fixed to 2 for further experiments using all Zol-containing nanocomplexes (Zol:HECP2k:pDNA or siRNA= 2:50:1, *w*/*w*/*w*).

### 3.9. Cellular Uptake Analysis with Flow Cytometry

The cellular uptake efficiency of the nanocomplexes was assessed with flow cytometry in RAW264.7 cells. YOYO-1 iodide-labeled pDNA was used for the formation of complexes. HECP2k polyplexes and HECP2k/(pDNA + Zol) nanocomplexes showed similar cellular uptake efficiency (88.5% and 89.1%, respectively), which was higher than that of PEI25k polyplexes (75.4%) (Figure S6). This means that the Zol-containing formulation did not have any negative effects on the cellular uptake of the nanocomplexes, maintaining similar sizes and Zeta-potential values with HECP2k polyplexes.

### 3.10. Serum Stability Test of the Nanocomplexes

The serum stability of HECP2k/(pDNA + Zol) nanocomplexes was evaluated by luciferase transgene assay on RAW264.7 cells in 30% FBS condition. As shown in Figure S7, the transfection efficiency of PEI25k polyplexes significantly decreased even in 10% serum condition, and it shrunk as cell value in 30% FBS condition due to the strong nonspecific interaction with serum proteins. However, in contrast to PEI25k polyplexes, the transfection efficiency of HECP2k/(pDNA + Zol) nanocomplexes remained almost the same even if FBS concentration increased from 10% to 30%, and it was more than 600 times higher than that of PEI25k polyplexes. The serum stability of HECP2k/(siRNA + Zol) nanocomplexes was also examined by measuring size changes in serum condition. During 5 h of incubation, they did not show any significant change, maintaining 150~180 nm sizes (Figure S8).

Therefore, it was concluded that Zol-containing nanocomplexes formulation did not have any negative effects on innate high serum stability of HECP2k polyplexes with hydrophilic and mobile ethylene oxide moieties preventing serum proteins from adhering to the complexes [31].

### 3.11. RANKL-Induced Osteoclast Differentiation of RAW264.7 Cells

Osteoclasts were obtained by differentiation of RAW264.7 cells by treatment of RANKL (100 ng/mL) every other day (Figure S9A). On the 7th day of culture, the differentiation of RAW264.7 cells into osteoclasts was identified by TRAP staining assay. TRAP is a specific factor that is expressed in osteoclasts [32]. As shown in Figure S9B, during the culture period, the purple color-staining of expressed TRAP began to appear on day 3, and the characteristic multinucleated osteoclasts started to be observed on day 5 of the culture. Therefore, it was confirmed that the RAW264.7 cells could be successfully differentiated into osteoclasts by RANKL treatment.

### 3.12. Osteoclast Differentiation Inhibition Analysis by TRAP Assay

**Figure 6:**
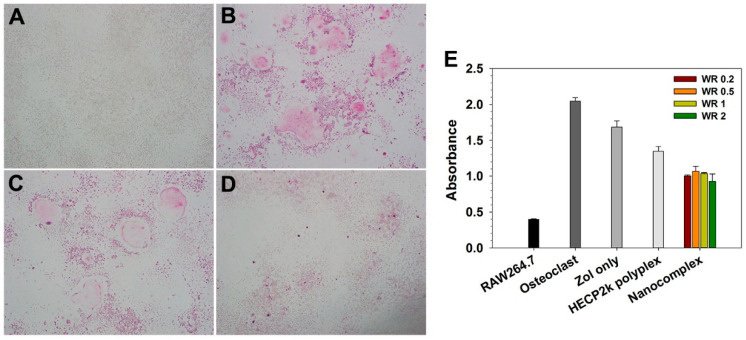
Osteoclast inhibition analysis by TRAP staining assay. (**A**) RAW264.7 cells, (**B**) osteoclasts differentiated from RAW264.7 cells by RANKL treatment, (**C**) Zol only-treated osteoclasts, and (**D**) HECP2k/(RANK siRNA + Zol) nanocomplexes-treated osteoclasts. (**E**) TRAP quantification result by measuring the absorbance of TRAP at 405 nm (WR means Zol/siRNA).

To confirm the inhibition of osteoclast differentiation by HECP2k/(RANK siRNA + Zol) nanocomplexes, TRAP staining and quantification were performed after the nanocomplexes were treated in the process of osteoclast differentiation. As shown in Figure 6B, RANKL-treated RAW264.7 cells showed purple-color stained and large multinucleated cell morphology after TRAP staining, meaning RAW264.7 cells were well differentiated to osteoclasts. Zol-treated cells showed somewhat decreased staining results and less multinucleated cell morphology, meaning Zol could inhibit the differentiation a little bit (Figure 6C). However, it was difficult to find any distinct staining and multinucleated cell morphology after HECP2k/(RANK siRNA + Zol) nanocomplexes were treated, which shows that the nanocomplexes could induce a strong inhibition effect on the TRAP expression and osteoclasts differentiation (Figure 6D).

Inhibition of osteoclast differentiation by HECP2k/(RANK siRNA + Zol) nanocomplexes was further quantified by TRAP assay (Figure 6E). RANKL-treated RAW264.7 cells showed about 5 times higher TRAP expression level than RAW264.7 cells, indicating their differentiation to osteoclasts. The nanocomplexes-treated cells showed the lowest absorbance, which was half the value of osteoclast control. Neither Zol only nor HECP2k/RANK siRNA polyplexes showed as much inhibition effect as the nanocomplexes. Therefore, it was proved that the HECP2k/(RANK siRNA + Zol) nanocomplexes possess a high capability of inhibiting osteoclast differentiation.

### 3.13. Induction of Osteoclast Apoptosis by HECP2k/(siRNA + Zol) Nanocomplexes

**Figure 7:**
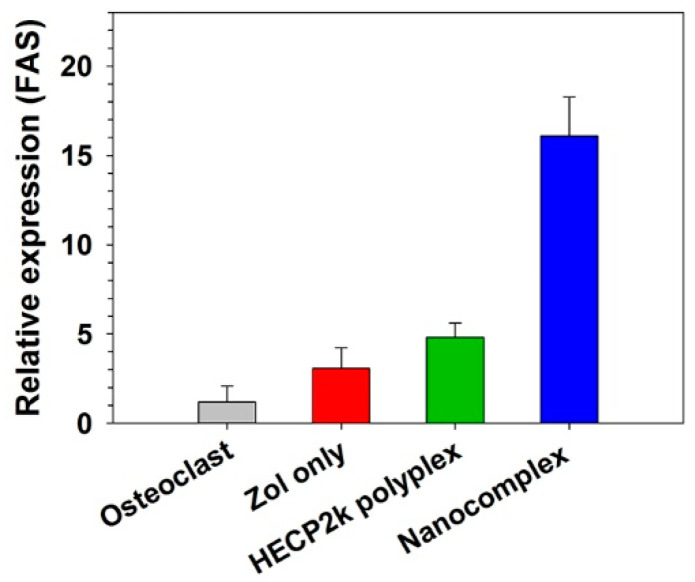
Relative FAS mRNA levels of HECP2k/(siRNA + Zol) nanocomplexes-treated osteoclasts by qPCR. Zol only and HECP2k/siRNA polyplexes were used as controls. All values were normalized to the value of non-treated osteoclasts.

The apoptosis-inducing effect of HECP2k/(siRNA + Zol) nanocomplexes was examined by qPCR using the apoptosis marker FAS gene [33]. Osteoclasts differentiated from RAW 264.7 cells were treated with Zol only, HECP2k/siRNA polyplexes, and HECP2k/(siRNA + Zol) nanocomplexes. As shown in Figure 7, it was found that Zol only and HECP2k polyplexes could induce just less than 5 times higher FAS gene expression than the normal level. Zol treatment did not show efficient apoptotic activity in osteoclasts, displaying its poor bioavailability when administered without proper carriers. However, the highest FAS gene expression was observed in the nanocomplexes-treated cells, which was about 16 times higher than the normal level. This result means that the nanocomplexes could induce a high level of apoptosis to osteoclasts via the combinatorial effect of RANK siRNA and Zol, which possess both endosome buffering and osteoclast inhibitory activity after high cellular uptake.

### 3.14. Inhibition of Osteoclast Activity of HECP2k/(siRNA + Zol) Nanocomplexes

**Figure 8:**
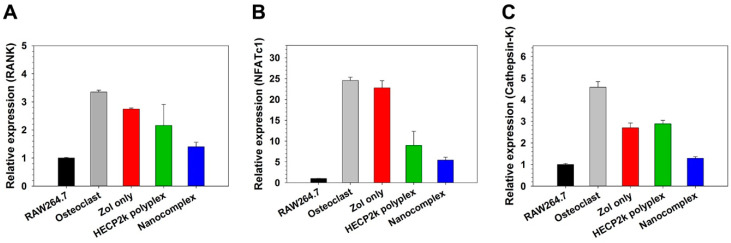
Relative mRNA levels of (**A**) RANK, (**B**) NFATc1, and (**C**) cathepsin-K for HECP2k/(siRNA + Zol) nanocomplexes-treated osteoclasts by qPCR. Zol only and HECP2k/siRNA polyplexes were used as controls. All values were normalized to the value of non-treated RAW264.7 cells.

To confirm the ability of the nanocomplexes to inhibit the osteoclast activity, the complexes were treated during the osteoclast differentiation of RAW264.7 cells and the osteoclast marker gene expression levels were examined by qPCR (Figure 8). RANK [34], NFATc1 [35], and cathepsin-K [36] gene expression levels were found to be highly increased in osteoclasts in comparison with RAW264.7 cells. After treatment of Zol only or HECP2k/siRNA polyplexes, the cells showed somewhat reduced gene expression levels. However, when the HECP2k/(siRNA + Zol) nanocomplexes were treated, the marker gene expression levels were dramatically reduced even to the levels of RAW264.7 cells, which means that the nanocomplexes could inhibit the activity of osteoclasts very efficiently. It is also thought that Zol can facilitate the endosomal escape of the nanocomplexes by endosome buffering, thus leading to a high inhibition effect of the osteoclast activity by RANK siRNA, with its own apoptosis-inducing and anti-resorptive effect together.

## 4. Conclusions

In this study, two anti-bone resorptive agents, RANK siRNA and Zol, were co-delivered by the nanocomplexes based on biocompatible HECP2k polymer for osteoclast inhibition. RANK siRNA and zoledronate could be easily complexed with HECP2k, forming HECP2k/(RANK siRNA + Zol) nanocomplexes. These nanoparticles have advantages in the fabrication of pharmaceutical agents into stable nanocomplexes via a simple method without complicated chemical conjugation. It was confirmed that both HECP2k/(pDNA + Zol) and HECP2k/(siRNA + Zol) nanocomplexes have controlled sizes and surface charges suitable for efficient cellular uptake, with spherical morphology. The nanocomplexes possessed high gene condensing ability as well as Zol loading efficiency, demonstrating the high feasibility for efficient gene/drug co-delivery systems. The HECP2k/(pDNA + Zol) nanocomplexes had not only low cytotoxicity but also higher gene transfection efficiency than PEI25 polyplexes or even HECP2k polyplexes, probably due to the buffering capacity of Zol that facilitated the endosomal escape of the complexes, augmenting the delivery efficiency. Therefore, it was suggested that Zol can function as both an antiresorptive drug and an agent for increasing gene delivery efficiency through endosomal escape at the same time. In addition, these particles showed high cellular uptake efficiency as well as serum stability without any deleterious effect in the presence of Zol. Based on the appropriate physicochemical properties of the nanocomplexes, HECP2k/(siRNA + Zol) nanocomplexes were finally utilized for osteoclast inhibition. The treatment of HECP2k/(RANK siRNA + Zol) nanocomplexes could inhibit the osteoclast differentiation and suppress the osteoclast activity efficiently, inducing apoptosis via the combinatorial effect of RANK siRNA and Zol. Consequently, it is concluded that HECP2k/(RANK siRNA + Zol) nanocomplexes prepared by simple mixing showed great potential for the treatment of osteoporosis through osteoclast inhibition.

## Data Availability

The original contributions presented in this study are included in the article/Supplementary Material. Further inquiries can be directed to the corresponding author.

## Supplementary Materials

The following supporting information can be downloaded at: https://www.mdpi.com/article/10.3390/pharmaceutics16121623/s1. Figure S1. ^1^H NMR spectrum (A) and GPC chromatogram (B) of HECP2k. Figure S2. Characterization of HECP2k(siRNA + Zol) nanocomplexes. (A) Agarose gel electrophoresis result. (B) Average size measurement result. (C) Zeta potential measurement result. Figure S3. TEM images of HECP2k/(siRNA + Zol) nanocomplexes (Zol:HECP2k:siRNA = 2:50:1, *w*/*w*/*w*). The morphology of the nanocomplexes was observed with transmission electron microscopy (JEM-2100PLUS, JEOL, Tokyo, Japan). 10 μL of complex solutions (2 μg siRNA) were loaded on a copper carbon film 300 mesh for 1 min. After blotting away the excess solution on the grid with a filter paper, the grid was stained for 5 s with 2% uranyl acetate solution. After blotting the grid away again, the images were visualized with an accelerating voltage of 200 kV. Figure S4. (A) Absorbance measurement results for HECP2k(pDNA + Zol) nanocomplexes (Zol:HECP2k:pDNA = 20:50:1, *w*/*w*/*w*) from 200 nm to 340 nm. No peaks were observed at over 340 nm. (B) Calibration curve of Zol prepared with the absorbance at 215 nm. Figure S5. (A) Acid-base titration curve for Zol. 0.1 M NaOH was used for titration. (B) Acid-base titration curve for each polymer and complex. All samples were titrated from pH 11 to pH 3. 0.1 M HCl was used for titration. Endosomal pH range (from 7.4 to 5.1) is marked with dotted lines. Figure S6. Cellular uptake analysis of (A) untreated RAW264.7 cells, (B) PEI25k polyplexes, (C) HECP2k polyplexes, and (D) HECP2k(pDNA + Zol) nanocomplexes by flow cytometry. Figure S7. Serum stability assessment of each complex by luciferase transgene expression assay. Transfection experiments were performed on RAW264.7 cells in 10% and 30% serum-containing media, respectively. Figure S8. Size change measurement of HECP2k(siRNA + Zol) nanocomplexes in 10% serum condition according to the incubation time (0~5 h). The Z-average sizes of HECP2k/(siRNA + Zol) nanocomplexes were measured by Zetasizer. The measurement was performed 3 times. Figure S9. (A) Scheme of RANKL-induced osteoclast differentiation from RAW264.7 cells. (B) Osteoclast differentiation from RAW264.7 cells by TRAP assay according to treatment time. Scale bar: 100 μm. Table S1. qPCR primer sequences for mRNA level quantification. Table S2. The polydispersity index (PDI) from the size measurements of the nanocomplexes.
