# Supplementary material for: Cationic Hydroxyethyl Cellulose Nanocomplexes and RANK siRNA/Zoledronate Co-Delivery Systems for Osteoclast Inhibition
Source: Pharmaceutics. 2024 Dec 22;16(12):1623. doi: 10.3390/pharmaceutics16121623 (PMC11679713; doi:10.3390/pharmaceutics16121623)
Supplement: Supplementary file 1 [file pharmaceutics-16-01623-s001.zip › pharmaceutics-3359067-supplementary.pdf]

Supplementary Materials

Cationic Hydroxyethyl cellulose Nanocomplexes and RANK siRNA/zoledronate Co-delivery Systems for Osteoclast Inhibition

Sohyun Lee[1], Seoyeon Park[1], Tae-il Kim[1,2,*]

[1] Department of Agriculture, Forestry and Bioresources, College of Agriculture and Life Sciences, Seoul National University, 1 Gwanak-ro, Gwanak-gu, Seoul 08826, Republic of Korea

[2] Research Institute of Agriculture and Life Sciences, Seoul National University, 1 Gwanak-ro, Gwanak-gu, Seoul 08826, Republic of Korea

* Correspondence: seal1004@snu.ac.kr

S1. ^{1}H NMR spectrum (A) and GPC chromatogram (B) of HECP2k.

A.

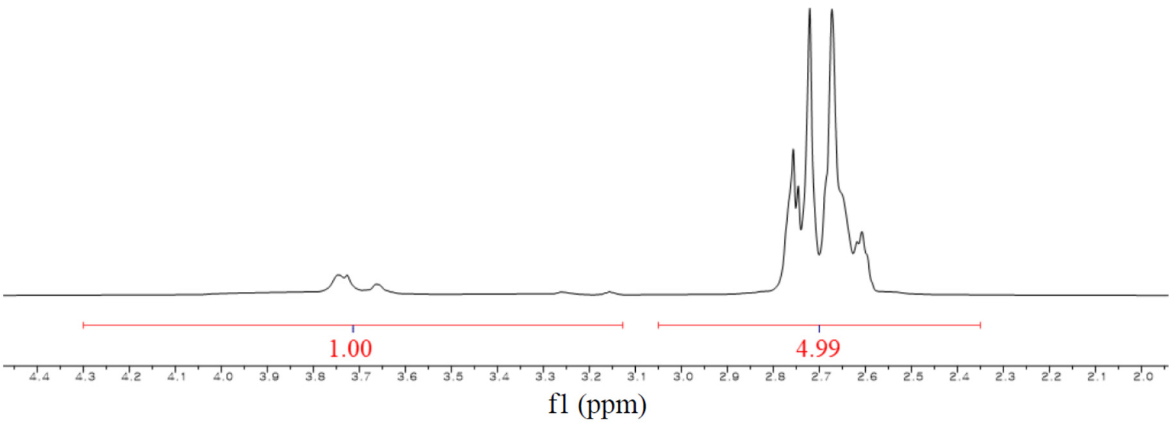

B.

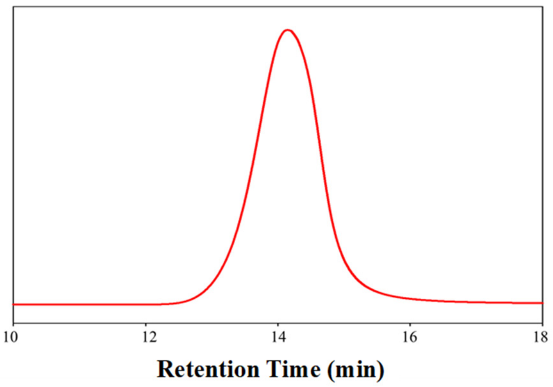

S2. Characterization of HECP2k(siRNA+Zol) nanocomplexes. (A) Agarose gel electrophoresis result. (B) Average size measurement result. (C) Zeta potential measurement result.

A.

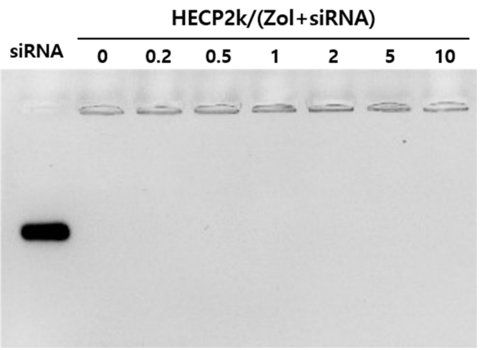

B.

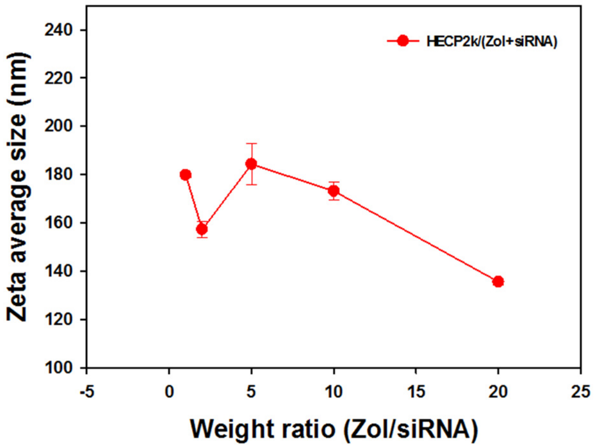

C.

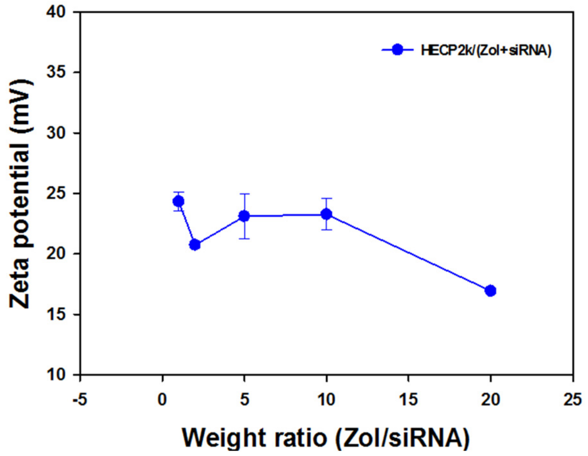

S3. TEM images of HECP2k/(siRNA+Zol) nanocomplexes (Zol:HECP2k:siRNA = 2:50:1, w/w/w). The morphology of the nanocomplexes was observed with transmission electron microscopy (JEM-2100PLUS, JEOL, Tokyo, Japan). 10 μL of complex solutions (2 μg siRNA) were loaded on a copper carbon film 300 mesh for 1 min. After blotting away the excess solution on the grid with a filter paper, the grid was stained for 5 s with 2% uranyl acetate solution. After blotting the grid away again, the images were visualized with an accelerating voltage of 200 kV.

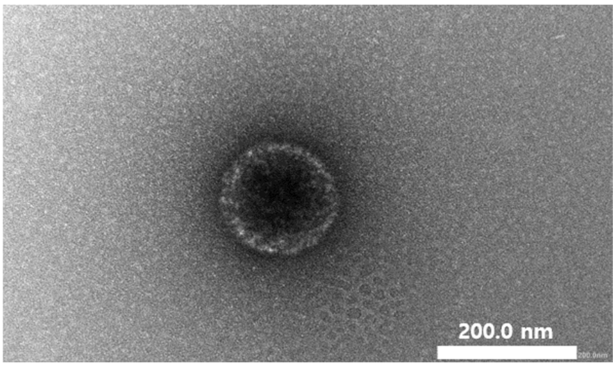

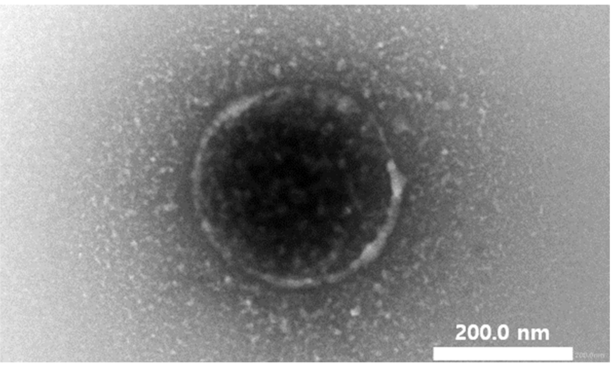

S4. (A) Absorbance measurement results for HECP2k(pDNA+Zol) nanocomplexes (Zol:HECP2k:pDNA = 20:50:1, w/w/w) from 200 nm to 340 nm. No peaks were observed at over 340 nm. (B) Calibration curve of Zol prepared with the absorbance at 215 nm.

A.

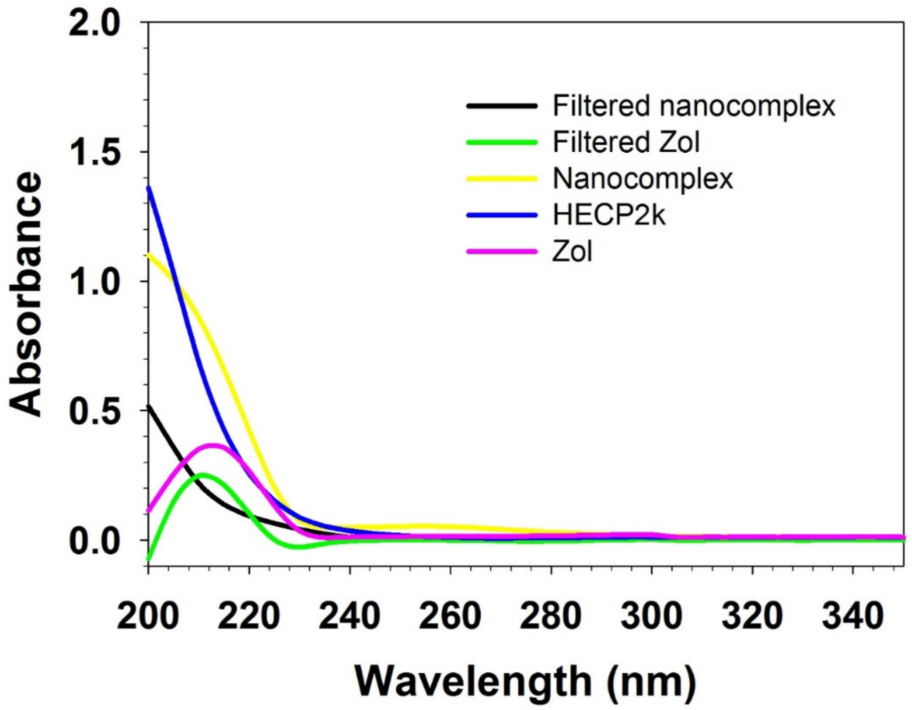

B.

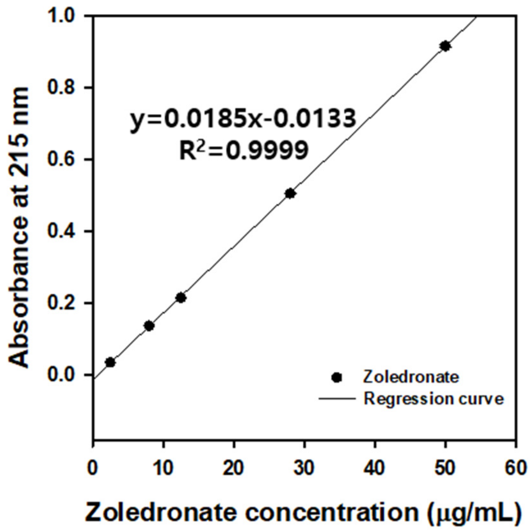

S5. (A) Acid-base titration curve for Zol. 0.1 M NaOH was used for titration. (B) Acid-base titration curve for each polymer and complex. All samples were titrated from pH 11 to pH 3. 0.1 M HCl was used for titration. Endosomal pH range (from 7.4 to 5.1) is marked with dotted lines.

A

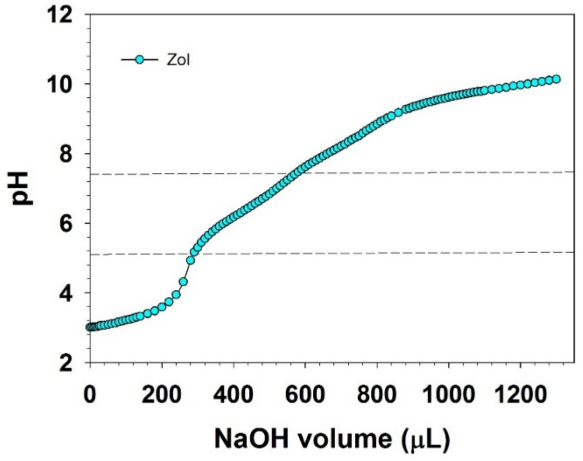

B

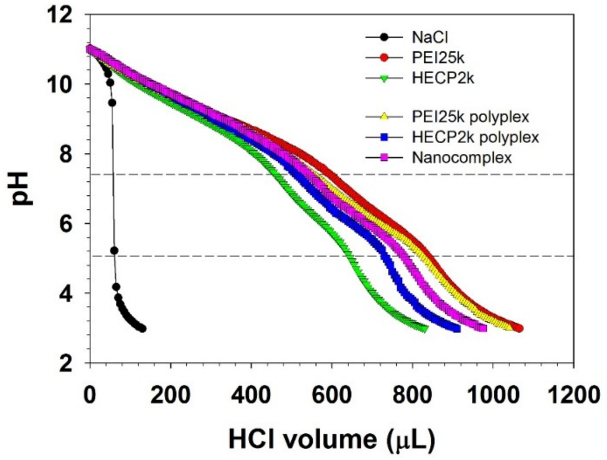

S6. Cellular uptake analysis of (A) untreated RAW264.7 cells, (B) PEI25k polyplexes, (C) HECP2k polyplexes, and (D) HECP2k(pDNA+Zol) nanocomplexes by flow cytometry.

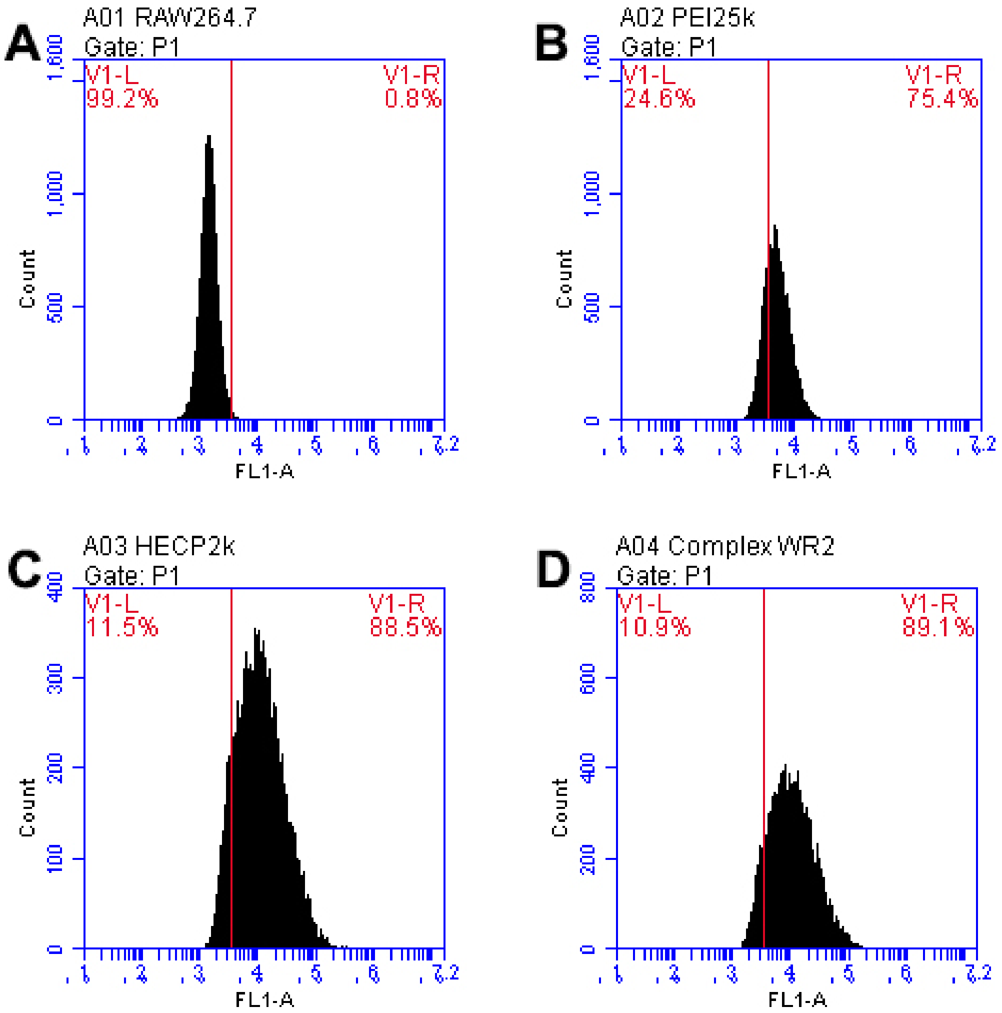

S7. Serum stability assessment of each complex by luciferase transgene expression assay. Transfection experiments were performed on RAW264.7 cells in 10% and 30% serum-containing media, respectively.

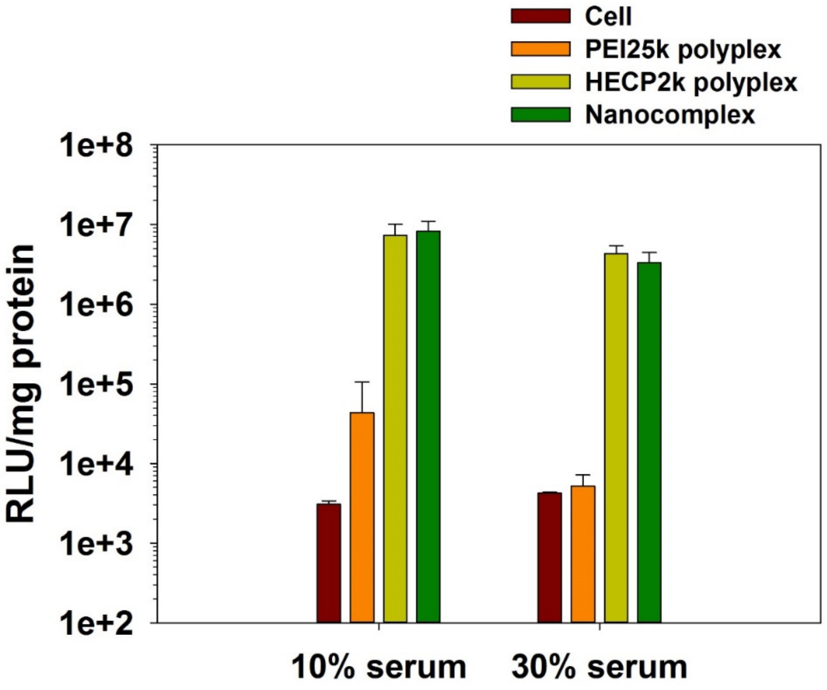

S8. Size change measurement of HECP2k(siRNA+Zol) nanocomplexes in 10% serum condition according to the incubation time (0~5 h). The Z-average sizes of HECP2k/(siRNA+Zol) nanocomplexes were measured by Zetasizer. The measurement was performed 3 times.

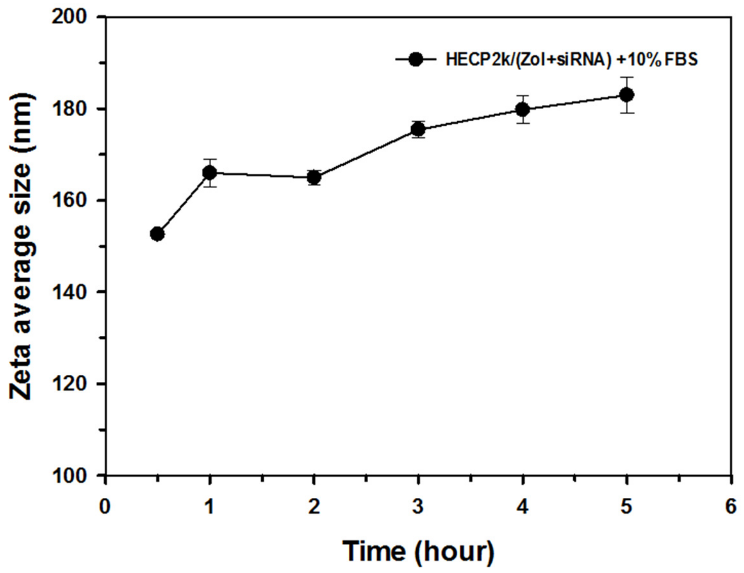

S9. (A) Scheme of RANKL-induced osteoclast differentiation from RAW264.7 cells. (B) Osteoclast differentiation from RAW264.7 cells by TRAP assay according to treatment time. Scale bar: 100 μm.

A

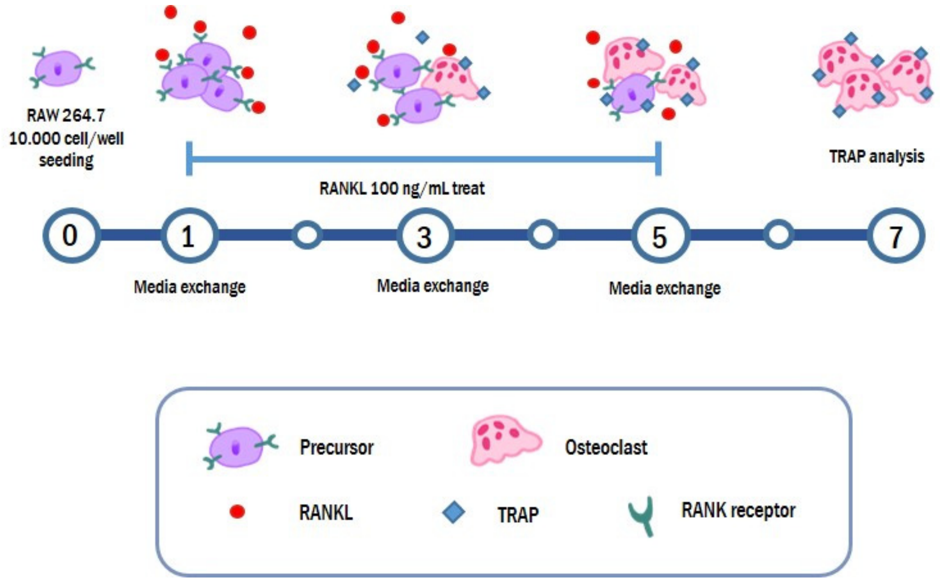

B

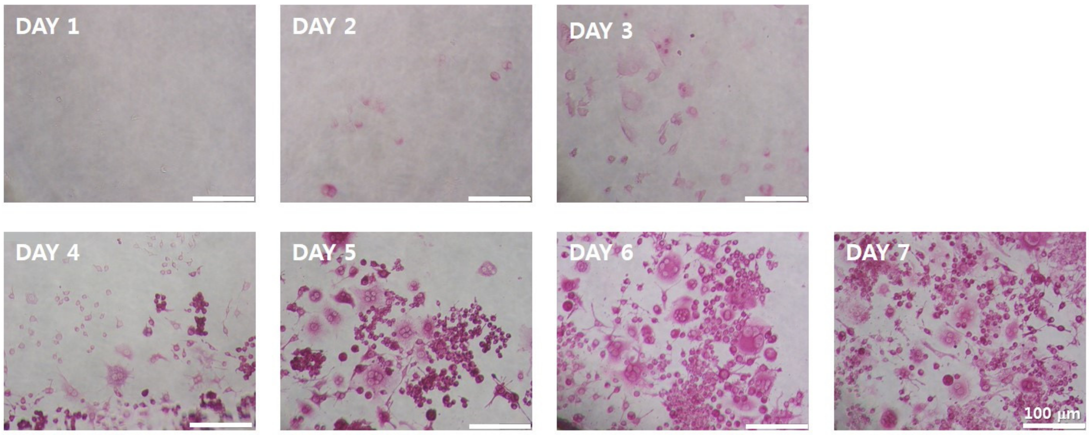

Table S1. qPCR primer sequences for mRNA level quantification.

Gene	Primer sequence (5'→3')	References
FAS	F-GGA GGC CCA TTT TGC TGT CAA CCA	[33]
	R-GTC CTT CTG GAC CAT GTC CTG	
RANK	F-AGA TGT GGT CTG CAG CTC TTC CAT	[34]
	R- ACA CAC TTC TTG CTG ACT GGA GGT	
NFATc1	F- TGC TCC TCC TCC TGC TGC TC	[35]
	R- CGT CCT CCA CCT CCA CGT CG	
Cathepsin-K	F-GCC AGG ATG AAA GTT GTA TG	[36]
	R-CAG GCG TTG TTC TTA TTC C	

Table S2. The polydispersity index (PDI) from the size measurements of the nanocomplexes.

Weight ratio (Zol/gene)	HECP2k(pDNA+Zol) nanocomplexes	HECP2k(siRNA+Zol) nanocomplexes
1	0.152	0.158
2	0.154	0.139
5	0.192	0.144
10	0.254	0.173
20	0.249	0.135
